# L-OPU in Goat and Sheep—Different Variants of the Oocyte Recovery Method

**DOI:** 10.3390/ani10040658

**Published:** 2020-04-10

**Authors:** Jarosław Wieczorek, Jurij Koseniuk, Maria Skrzyszowska, Mirosław Cegła

**Affiliations:** 1 University Centre of Veterinary Medicine UJ-UR, University of Agriculture in Krakow, 30-059 Krakow, Poland; 2 Artvimed Centre for Reproductive Medicine, 30-147 Krakow, Poland; jerzy.koseniuk@artvimed.pl; 3 Department of Reproductive Biotechnology and Cryoconservation, National Research Institute of Animal Production, 30-083 Balice/Krakow, Poland; maria.skrzyszowska@izoo.krakow.pl (M.S.); miroslaw.cegla@izoo.krakow.pl (M.C.)

**Keywords:** laparoscopy, ovum pick up, oocyte, goat, sheep

## Abstract

**Simple summary:**

In this study, minimally invasive laparoscopic methods of recovering oocytes in goat and sheep (L-OPU, laparoscopic ovum pick-up) were developed and improved. Applying the laparoscopic technique allows animal welfare to be significantly improved while still maintaining high effectiveness of the method. The method allows a high number of good quality oocytes to be obtained and additionally reduces the invasiveness of the method and tissue damage which occurs during the operation to a minimum. It requires a short time and allows animals to return to the herd immediately after the operation has been completed. Additionally, it also gives the opportunity for maximum use of the animal’s genetic capability. The limitation of using the L-OPU method in goats is its variable and changing effectiveness. That is why it is crucial to develop an effective and repeatable method. The study compared several variants of the method including varying techniques of conducting the operation, various tools for recovering oocytes, and different plans of hormonal stimulation. As a result, an optimal method of recovering oocytes was developed. The research may be of great importance in improving the welfare of animals and increasing the effectiveness of biotechnological methods used in goat and sheep breeding as well as in the area of research.

**Abstract:**

The laparoscopic method of recovering oocytes in goats and sheep is one of the minimally invasive methods used in the biotechnology of animal reproduction. It allows for good quality oocytes that are suitable for in vitro maturation and fertilization to be recovered. The limitation of using the laparoscopic ovum pick-up (L-OPU) method in goat and sheep is its changing effectiveness and the lack of repeatability of results, as well as the varying effectiveness of different variants of the method. Therefore, it is necessary to develop effective non-invasive techniques allowing for multiple good quality oocyte recovery that would be suitable for in vitro maturation and fertilization. In this study, four different L-OPU variants were described in goats and sheep. Various techniques of recovering oocytes were discussed, including the techniques of conducting the operation, various tools for recovering oocytes, and different plans of hormonal stimulation. Recovery rates were 35% (Variant I), 57% (Variant II), 72% (Variant III), and 67% (Variant IV). After evaluation, 94% (both Variant I and II), 93% (Variant III), and 84% (Variant IV) of the oocytes were qualified for in vitro maturation. The results of the study show that the proposed technique of laparoscopic recovery of oocytes allows a sufficient number of ovarian cells suitable for in vitro culture to be obtained and as a consequence it makes them useful in in vitro maturation/in vitro fertilization (IVM/IVF) programs or cloning. The method allows for a fast and effective conduct of the operation in a living donor with minimal invasiveness while preserving the excellent condition of animals.

## 1. Introduction

Endoscopic methods are used in breeding animals for recovering and transferring embryos, for intra-uterine insemination, and for diagnostic purposes, e.g., ovary and uterus evaluation, ovulation observation and development of corpus luteum, and oocyte recovery [1,2,3]. An increasing interest in non-surgical methods of oocyte recovery, as well as recovery and transfer of embryos for research purposes and practical implementation can be seen [4]. This results from the intention to maximize the application of the genetic potential of the animals on the basis of modern biotechnological programs: estrus synchronization, superovulation, oocyte recovery, in vitro maturation and fertilization, cryopreservation, and embryo transfer [5,6,7]. With the varying responses to superovulation [8] and the effectiveness of methods of obtaining and transferring embryos ranging between 22% and 80% [3,5,9], maturation and in vitro fertilization, and the production of embryos, have become an attractive option used in research and in farm animals. In ewes and goats, methods based on the MOET program (Multiple Ovulation Embryo Transfer) have been introduced [10]. IVM/IVF (in vitro maturation/in vitro fertilization) programs have been developed for the routine production of small ruminant (sheep and goats) embryos [11,12]. The majority of oocytes used for research are recovered from ovaries obtained post-slaughter. Oocytes are recovered from living donors by aspiration of ovarian follicles using surgical methods which cause traumatization and limit the possibility of multiple use of the same animals [13]. Other methods of obtaining oocytes from living donors are laparoscopic minimally invasive techniques, but these are less effective [13]. In medium-sized farm animals, the OPU (ovum pick-up) technique can be used along with a video surgery technique which enables almost bloodless and fast access to abdominal organs, simple ovary stabilization, and aspiration of ovarian follicles [14]. The effectiveness of the method depends on the breed and individual features of the animals, efficient synchronization and superovulation, pre-op and surgical procedures, and the experience of the surgeon (the person conducting the procedure) [14,15]. A comparison of laparoscopic ovum pick-up (L-OPU) results in goats and sheep obtained by different authors shows that the limitation of this method is its varying effectiveness, lack of reproducibility of results, and varying efficacy in different variants of the method [1,16,17]. The aim of this study was to develop a non-invasive and effective technique that would allow multiple recovery of high-quality oocytes which would make them suitable for in vitro maturation and fertilization.

## 2. Materials and Methods

### 2.1. Animals

All procedures were performed with the prior approval of II Animal Ethics Committee in Cracow, approval number 453/2007. The oocyte donors were 88 (*n* = 88) goats of the Alpine breed, aged 1–3 years, bodyweight around 45–50 kg, and 45 (*n* = 45) ewes of the Wrzosówka (Polish Heath Sheep) breed, bodyweight around 35–40 kg, aged 1–3 years. The goats and sheep were from the same herd. Oocytes were recovered only one time from one donor; no donor was used more than one time.

### 2.2. Procedure

**Figure 1:**
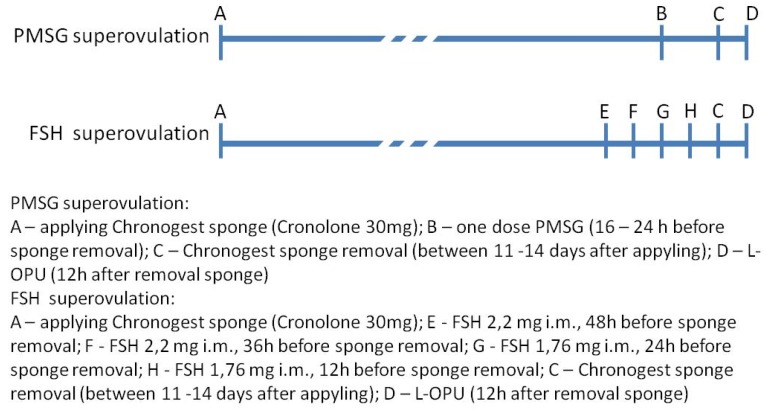
Schedule of estrus synchronization with Cronolone and superovulation with pregnant mare serum gonadotropin (PMSG) or follicle-stimulating hormone (FSH) treatment.

Four goat protocols were compared (4 variants) for the L-OPU method. Goat oocytes were obtained using 4 variants of the method during 17 subsequent recovering sessions. Sheep oocytes were obtained in one variant during 6 sessions. The number of animals in groups and the order of sessions were as followed: Variant I—10 goats (sessions 1–3); Variant II—10 goats (sessions 4–6); Variant III—43 goats (sessions 7–13); Variant IV—25 goats (sessions 14–17). Ewe oocytes were recovered using one variant of the method (Variant III—45 sheep in 6 sessions). In each session, oocytes from 3–10 animals were obtained. All the donors were subjected to estrus cycle synchronization and superovulation according to the schedule shown in Figure 1. Estrus cycle synchronization in all donors (goats and sheep) was carried out using the intravaginal sponges Chronogest (Cronolone 30 mg, MSD Animal Health GmbH, Igoville, France) lasting for 11–14 days. Superovulation was induced in two ways. The first method was a single treatment of the animals with one dose of 1000 IU PMSG (pregnant mare serum gonadotropin) (Folligon, MSDAnimal Health GmbH, Brussels, Belgium) from 16 to 24 h prior to sponge removal; oocytes were recovered 24 h after removing the sponges. The first superovulation protocol was used in the goats (*n* = 63) in the I, II, and III OPU variants of the OPU method and in all the sheep. The second method for inducing superovulation was with four applications of FSH (follicle-stimulating hormone) (Ovagen, ICPbio LTD., Auckland, New Zealand) according to the schedule shown in Figure 1. Sponges were removed 12 h after the last application of FSH. Oocytes were recovered 12 h after removing sponges. This superovulation scheme was conducted only in the IV OPU variant (25 goats).

In all animals, the same method of general anesthesia was used. Prior to the operation, the animals were premedicated with atropine 0.05 mg/kg i.m. (Atropinum Sulfuricum 0.5 mg, Polfa Warsaw, Poland) and xylazine 3–5 mg/kg i.m. (Sedazin, Pulawy, Poland). General anesthesia was induced with thiopentone natrium 5–10 mg/kg i.v. (Tiopental 1.0 g, Sandoz GmbH, Kundl, Austria). The time of anesthesia varied from 15 to 30 min. The time of operation was measured from the moment of inducing anesthesia with thiopentone to the moment of finishing the last skin suture.

**Figure 2:**
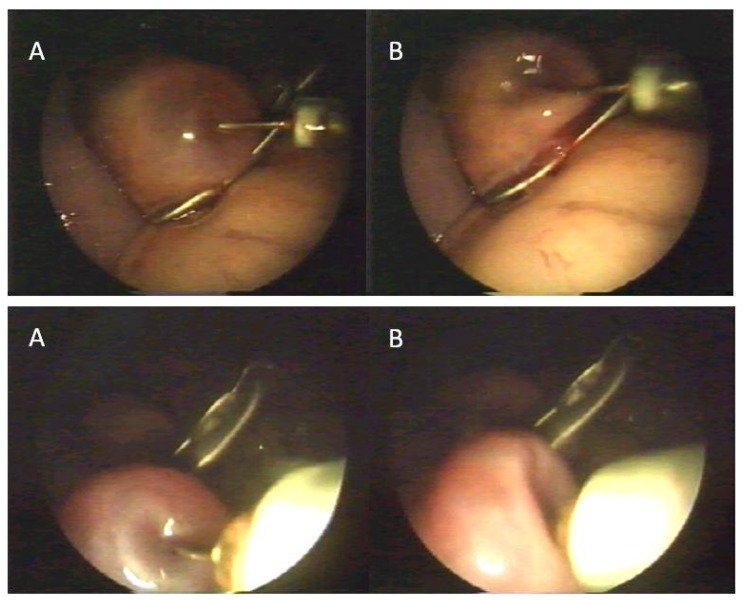
Oocyte recovery—aspiration of fluid from ovarian follicles with oocytes. (**A**)—follicle before aspiration, (**B**)—follicle after aspiration.

After positioning the animal in dorsal recumbency, an endoscope was inserted into the abdominal cavity through the umbilicus. After filling the abdomen cavity with filtered air, two trocars for placing the manipulators were inserted 15 cm cranial to the udder. The trocars were inserted on an isosceles triangle plan, with sides of 15–20 cm. Afterwards, grasp forceps were inserted into the abdominal cavity for ovary stabilization. The ovary, after being isolated from the infundibulum, was stabilized by holding the ovarian ligament as close to its end as possible. After stabilizing the ovary, a catheter for oocyte aspiration was inserted through a second trocar. Oocytes were recovered in four variants of the method. In Variant I and II, an endoscope camera was inserted at the abdominal midline, grasp forceps were inserted to the left of the midline, the trocar for introducing the syringe for oocyte aspiration was inserted to the right of the midline. Oocytes were obtained by aspiration of fluid from ovarian follicles with oocytes (Figure 2).

**Figure 3:**
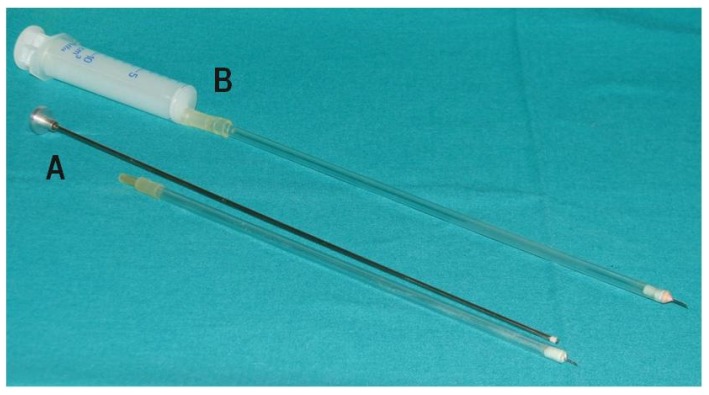
Tools for oocyte aspiration. A—originally designed 1 mL syringe for oocyte aspiration with a needle (needle: 1 cm long and 0.6mm diameter, 23 G; Polish Patent Application P-361068, 2003) (Variants I and II), B—catheter for oocyte aspiration (Variants III and IV).

At the beginning (Variant I), oocytes were recovered by aspirating with an oocyte aspiration pump (Vetec Aspirationpumpe, Mariensee, Germany). Due to the low effectiveness of this method, Variant II was applied in which the device for oocyte recovery was replaced with an originally designed 1 mL syringe for oocyte aspiration (Figure 3A). In this variant, depending on follicle size, 1 to 8 follicles were aspirated per ovary in a single attempt. To increase effectiveness, Variant III was applied in which the positions of the endoscope camera and the left grasp forceps were interchanged and the aspiration syringe was replaced by the catheter with a 10 mL syringe (Figure 3B). To get optimal results, Variant IV was applied in which the method of oocyte recovery was maintained as in Variant III but a different method of inducing superovulation was used. Taking into account the results obtained in Variants I–IV in goats, Variant III was considered to be the best and it was Variant III that was used for recovering oocytes from ewes.

In all variants of the method, the catheters were removed in reverse order after oocyte recovery. Neither the peritoneum nor abdominal walls needed to be sutured due to a minimal wound size of 0.5 to 1 cm; single simple absorbable sutures were placed into the skin. After the operation, the animals were observed at their stalls for 7 days. Analgesic drugs, meloxicam 1–2 mg/kg (Metacam 5%, Boehringer Ingelheim Vetmedica GmbH, Rhein, Germany), were applied once just after the operation. They were not used anymore during the remaining period of observation due to the lack of indications.

**Figure 4:**
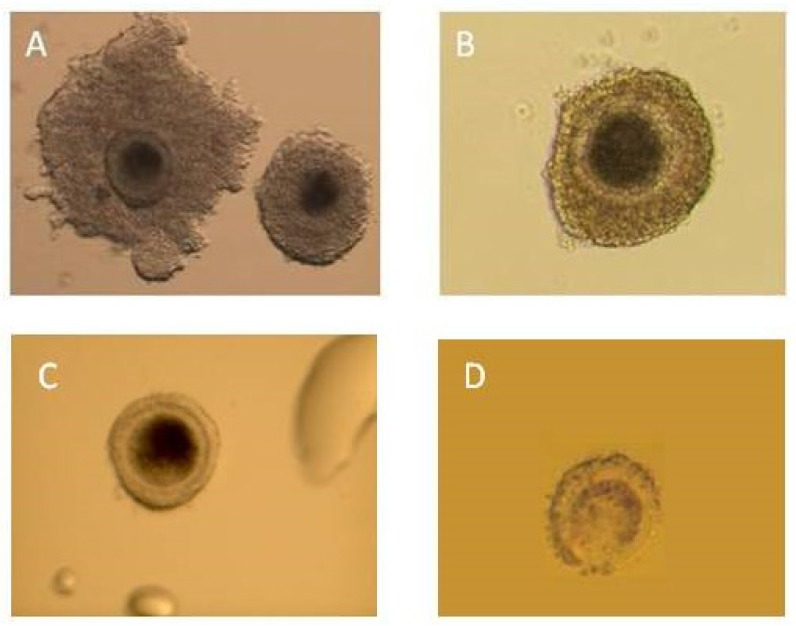
Immature oocytes collected using the ovum pick-up technique. (**A**) Class I—excellent quality of cumulus–oocyte complexes (COCs); morphologically normal oocytes with evenly granulated (homogenous) cytoplasm, surrounded by thick compact layers of cumulus cells; (**B**) class II—good quality COCs, morphologically normal oocytes with evenly granulated (homogenous) cytoplasm, surrounded by at least three compact layers of cumulus cells; (**C**) class III—cumulus cell-deprived oocytes (naked oocytes); (**D**) class IV—poor quality oocytes with clearly visible degenerative (necrotic or apoptotic) changes in cytoplasm.

Fluid containing oocytes were collected in a Petri dish containing Medium 199 HEPES Modification (Sigma-Aldrich, cat. no. M2520, Poznan, Poland) supplemented with heparin 0.5 IU/mL (Heparinum 5000 WZF IU/mL, Poland). This was flushed three times using the same medium supplemented with heparin, and subsequently it was evaluated using a stereoscopic microscope (25-40x). In the study, a four-degree scale of evaluation of oocytes was used. Quality changes in cytoplasm were accepted as a disqualifying factor regardless of the number of granulosa cells in the complex of oocytes. The following rules for the evaluation and classification of the oocytes were established: class I—homogenous cytoplasm, at least 3 layers of granulosa cells, class II—homogenous cytoplasm, 1–2 layers of granulosa cells, class III—homogenous cytoplasm, no granulosa cells, class IV—heterogenous cytoplasm, regardless of the number of granulosa cells. Oocytes classes I, II, and III were qualified for in vitro maturation (Figure 4).

### 2.3. Statistical Analyses

Statistical analyses were conducted using the Statistica 13.3 software (Tibco Statistica, Krakow, Poland). The data for goats and sheep were statistically analyzed using the one-way ANOVA test. The test was used for independent samples. The variables were treated as independent samples. In this way the influence of subsequent procedures on the number and quality of obtained oocytes was evaluated.

## 3. Results

**Figure 5:**
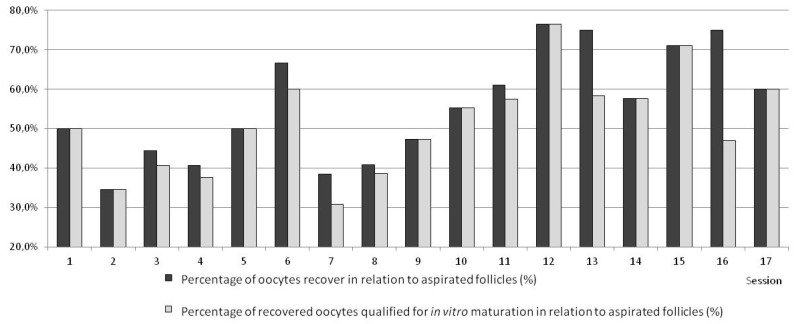
Comparing various L-OPU sessions with respect to percentages of recovered oocytes and oocytes selected for in vitro maturation (IVM) in sheep. (Variant I—sessions 1–3, Variant II—sessions 4–6, Variant III—sessions 7–13, Variant IV—sessions 14–17).

**Figure 6:**
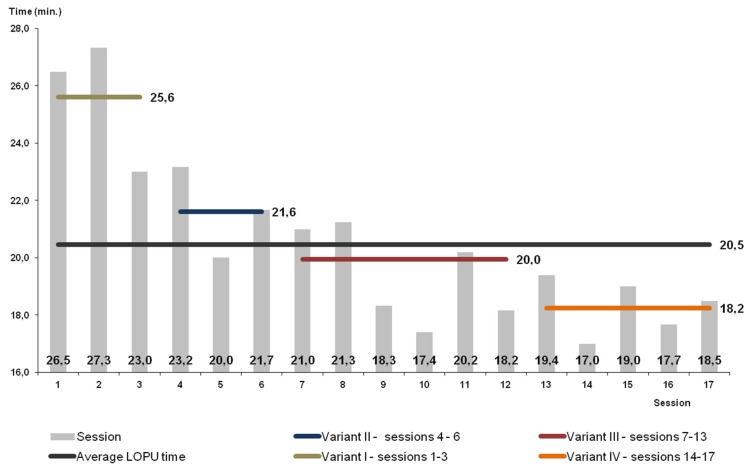
Timing of the L-OPU procedure in goats.

**Figure 7:**
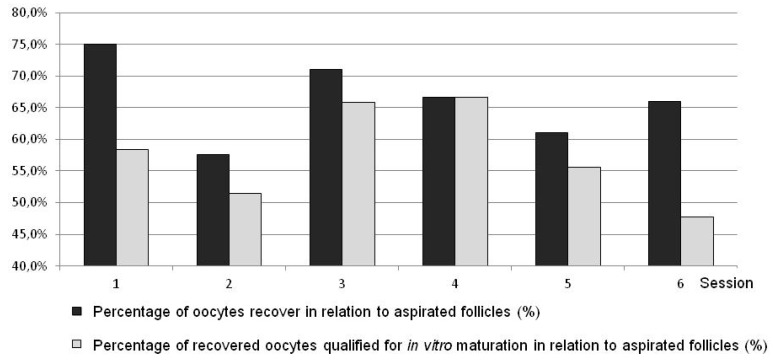
Comparing various L-OPU sessions with respect to percentages of recovered oocytes and oocytes selected for IVM in sheep.

**Figure 8:**
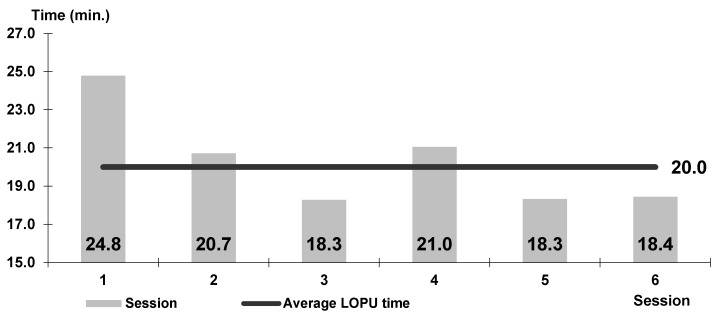
Timing of the L-OPU procedure in sheep in consecutive sessions.

The results are presented in Table 1 and Table 2 and in Figure 5, Figure 6, Figure 7 and Figure 8.

The highest effectiveness of superovulation was achieved in Variant I (the highest number of ovarian follicles per donor; Table 2). In this group, the quality of obtained oocytes was the best; altogether, 85% of them were qualified as classes I and II (<0.05). Despite the highest effectiveness of superovulation, this percentage of oocytes recovered and qualified for in vitro maturation was lower than in the other variants (*p* < 0.05). Due to the low efficacy in this variant, the method was modified. The statistically significantly lower efficacy in Variant I shows the high improvement of the method by the employment of proposed modifications of oocytes recovery in consecutive variants. After modifying, in Variant II, the percentage of recovered oocytes was higher than in Variant I (but without statistical significance and more oocytes were qualified for in vitro maturation and fertilization. However, the worsening of quality of obtained oocytes was observed. Despite the increase, effectiveness was still not sufficient in Variant II. Changes applied in Variant III increased the effectiveness of the method (Table 1). The number of recovered oocytes was highest (*p* < 0.05). In Variant III, the highest number of oocytes was classified for in vitro maturation in comparison with other variants (Figure 5). In Variant IV, a potentially more effective method of superovulation was tested. The high efficiency of FSH was proved. There were more follicles and recovered oocytes per single ovary but the difference was not statistically significant. The highest efficiency of superovulation was noticed in Variant I but high effectiveness of superovulation after applying PMSG was not proved in Variants II and III. At the same time, after FSH the quality of recovered oocytes was statistically significantly improved. In Variant III, a statistically significant (*p* < 0.05) higher number of class I oocytes was proved, as was a statistically significant (*p* < 0.05) lower number of class II oocytes, and a statistically significant (*p* < 0.05) higher number of class III oocytes and oocytes not qualified for in vitro maturation and fertilization. In Variant IV, a higher number of oocytes recovered from one donor (statistically significant; *p* < 0.05) was proved and a higher number of class I oocytes recovered from one donor (*p* < 0.05) was proved, while a lower number of class II oocytes recovered from one donor (*p* < 0.05) and a higher number of class III oocytes recovered from one donor (*p* < 0.05) were proved. There was no statistically significant difference in the number of not qualified oocytes recovered from one donor. The high standard deviation expressed per one donor indicates a lack of superovulation recurrence and significant individual diversity, similarly to PMSG and FSH superovulation. The time of the operation in goats was 15–30 min, on average 20.5 min, without statistically significant differences (Figure 6).

The results in ewes were comparable to the respective Variant III method in goats (Table 1, Figure 5 and Figure 7). In ewes, statistically more recovered oocytes were disqualified (Figure 5 and Figure 7). The results obtained in ewes were not compared to the results obtained in goats in Variants I, II, and IV.

The time of operation in ewes was 15–30 min, on average 20 min (Figure 8).

## 4. Discussion

The general procedure of the laparoscopic OPU method in goats and sheep gives the possibility of multiple recovery of oocytes from living donors in a minimally invasive way while preserving the welfare of animals. At the initial stage (Variant I), the assumed level of effectiveness was not achieved (insufficiently high number of recovered oocytes) while still preserving the high quality of aspirated ovarian cells. For this reason, the procedure was modified in Variant II. In Variant II, the method of inserting trocars and manipulators allowed easy access to ovaries and recovery of oocytes with a wide view of the covering abdominal organs and grasping forceps as well as the catheter. According to preliminary research carried out on pig organs in a simulator, stabilization of the ovary is optimal using a single pair of grasp forceps placed as close as possible to the end of mesosalpinx [18]. Admittedly, ovary stabilization with two pairs of grasp forceps guarantees more reliability but it requires the insertion of an additional, fourth trocar and made it impossible to operate the tools freely inside the abdominal cavity [14,18]. In this modification of the method, another tool for aspirating oocytes was used. For recovery of oocytes using the transvaginal OPU method in cattle, a variety of apparatus for aspirating follicular fluid are used [19,20]. The main advantage of that system is that it allows multiple flushings of the aspirated follicle. However, it was proved that during this procedure the mechanical denuding of the recovered oocytes occurs [21]. For this reason, in the current study the oocytes were aspirated with an originally designed syringe prepared for the recovery of oocytes or with a catheter used for aspiration. Aspiration with the syringe or catheter allowed the proper morphology of the oocytes to be maintained. The oocytes were initially aspirated with the syringe for the recovery of oocytes (sessions 1–6). Due to the small size of the syringe and the need to exchange it often, it was replaced with a catheter which allowed constant aspiration of follicular fluid. The implemented modifications led to a reduction in the time of operation of 3–5 min and increased its average effectiveness by about 60% (*p* < 0.05) (Figure 5 and Figure 7).

After FSH stimulation, a higher number of oocytes were recovered (there was no statistically significant difference) with higher quality ovular cells in comparison to PMSG stimulation. These results are comparable to the results obtained by other authors, more high-quality oocytes suitable for IVM/IVF were recovered after FSH stimulation [22,23]. In goats, after PMSG stimulation, the high number of large, non-ovulated follicles was observed, as well as a shortening of the estrus cycle and premature estrus. These phenomena result from the insufficiency of the large ovarian follicles as a response to the initial preovulatory increase in luteinizing hormone (LH) and high level of estrogen released from the unovulated follicle [23,24]. The earlier release of PGF2alfa was also observed in a case of occurrence of large follicles. In goats stimulated with PMSG, two atypical forms of corpus luteum were also observed. These were corpus rubrum (CH, corpus hemorrhagicum), which is present as a consequence of prolonged ovulation, and corpus luteum, prematurely regressing (at an early luteal phase) as a consequence of premature release of prostaglandin (PGF2alfa) [24,25].

The obtained results are comparable to the results of other authors (Table 3 and Table 4). In goats, 56% to 88% of aspirated oocytes were recovered. Moreover, 28.5% to 95.2% of those oocytes were qualified for in vitro maturation (Table 3). During our own research, a higher percentage of oocytes qualified for IVM/IVF was obtained in comparison to percentages obtained in the studies of other researchers. On average, 28.3% to 85.6% of aspirated oocytes were recovered in ewes and 65% to 86% of them qualified for in vitro maturation (Table 4). During our own research, sheep obtained a high percentage of qualified oocytes in comparison to percentages obtained in the studies of other researchers (Table 4). Kühholzer et al. [26], in their research on ewes which were not hormonally stimulated, obtained similar results—67.9% of aspirated oocytes were recovered with 83% of these qualifying for IVM/IVF. The same team, during an experiment on ewes subjected to synchronization of estrous cycle, obtained worse results—a recovery rate of 55.5% with 65% to 70% of the recovered ovular cells qualifying for IVM/IVF [14].

The mean time of surgery was 20 min, which was much shorter in comparison to the time described by Corodeiro et al. [27] (35 min) and Teixeira et al. [28] (26.75 ± 9.6 min) during the aspiration of ovarian follicles. These reports show that the laparoscopic technique allows to perform different kinds of operation in a short time, especially in small ruminants. The shortening of the time of surgery in consecutive sessions (1–17) was the result of the increasing skills and better cooperation of surgeons. These observations were also noticed by other authors [28,29].

The main criterion of quality was the preservation of the proper morphology of the recovered ovular cells and their ability to develop and be raised in vitro. There are different criteria used for evaluation and classification of oocytes, from two to six degrees [29,30,31,32,33,34,35]. The best quality oocytes are those with homogenous cytoplasm, with corona radiata and a thick layer of cells of cumulus oophorus (COC—cumulus oocyte complexes). Slightly worse quality oocytes may be qualified for IVM/IVF as well, including those with homogenous cytoplasm, with corona radiata and with an expanded layer of cells of cumulus oophorus (expanded COCs). There are some authors who qualify for IVM/IVF oocytes without granulosa cells (DCOCs, cumulus-denuded oocytes), with proper cytoplasm [29,30]. A similar evaluation and classification scale of oocytes was used by Koeman et al. [31] and Locatelli et al. [30]. In the present study, on average 50.41% of aspirated oocytes were recovered. During our own research, the authors of the article obtained a higher percentage of oocytes qualified for in vitro maturation in comparison to other researchers. Owing to the use of needles of proper size (20–22 G), short catheters of a length of 40 cm and lower pressure, mechanical damage to the corona radiata and the layer of cells of the cumulus oophorus was limited and as a consequence it was possible to improve the quality of recovered oocytes [13]. For follicle aspiration and recovery of at least good quality oocytes, the essential conditions are low pressure at the level of 0.2–0.4 Bar and medium sized needles of 20–22 G [36]. As the pressure is increased, the effectiveness of the recovery of oocytes and their quality rapidly decrease. In this case, the main change consists of the rising number of denuded oocytes. Admittedly, aspiration with a syringe does not allow the pressure to be monitored, but according to the obtained results and professional articles it can be assumed that this pressure varied within the suggested range of 0.2–0.4 Bar [36,37]. Additionally, in the chosen research model, a four-grade scale of evaluation was used. According to the scale, oocytes without granulosa cells were accepted for in vitro maturation. Such oocytes were not qualified by other authors using stricter criteria [30,32,33].

## 5. Conclusions

The proposed L-OPU variants have varying efficacy. During the study, an effective L-OPU method in sheep and goats was elaborated. The proposed method of laparoscopic obtaining of oocytes allows for a fast and effective procedure with minimal invasiveness which maintains the good health condition of animals. It is possible to effectively and quickly recover oocytes of good quality which may be used for in vitro culture. The good quality of obtained oocytes makes it possible to use the proposed procedure in IVM/IVF programs, in cloning, and in programs for the preservation of genetic resources using ex situ methods.

## Figures and Tables

**Table 1:** The effectiveness of the laparoscopic ovum pick-up (OPU) method among goats and ewes.

		Variant I (Goats)			Variant II (Goats)			Variant III (Goats)			Variants I to III in Total (Goats)			Variant IV (Goats)			Sheep		
		No.	A	B	No.	A	B	No.	A	B	No.	A	B	No.	A	B	No.	A	B
Number of ovarian follicles		98			66			246			410			202			212		
Number of aspirated follicles		96			65			238			399			195			205		
Total number of recovered oocytes		34	35.4 * ± 0.2		37	56.9 ± 0.3		139	72.1 * ± 0.2		210	52.6 ± 0.3		132	67.7 ± 0.2		137	66.8 ± 0.3	
Not qualified for IVM (Class IV)		2	2.1 ± 0.04	5.9 ± 0.1	2	3.1 ±0.07	5.4 ± 0.1	10	4.2 ± 0.1	7.2 ± 0.3	14	3.5 ± 0.1	6.7 ± 0.2	20	10.3 * ± 0.1	15.2 * ± 0.2	19	9.3 * ± 0.2	13.9 * ± 0.4
Qualified for IVM Total (1 + 2 + 3)		32	33.3 * ± 0.2	94 ± 0.1	35	53.8 ± 0.3	94.6 ± 0.1	129	54.2 ± 0.3	92.8 ± 0.3	196	49.1 ± 0.3	93.3 ± 0.2	112	57.4 ± 0.2	84.8 ± 0.2	118	57.6 ± 0.3	86.1 ± 0.3
Morphology oocytes qualified for IVM	Class I (1)	12	12.5 * ± 1.4	35.3 * ± 0.4	11	16.9 ± 0.2	29.7 ± 0.3	55	23.1 ± 0.3	39.6 * ± 0.4	78	19.5 ± 0.3	37.1 * ± 0.4	58	29.7 ± 0.2	43.9 * ± 0.3	44	21.5 ± 0.3	32.1 ± 0.4
	Class II (2)	17	17.7 ± 1.9	50.0 * ± 0.4	18	27.7 ± 2.0	48.6 * ± 0.4	52	21.8 ± 0.3	37.4 ± 0.4	87	21.8 ± 0.3	41.4* ± 0.4	16	8.2 * ± 0.2	12.1 * ± 0.3	58	28.3 ± 0.3	42.3 ± 0.4
	Class III (3)	3	3.1 ± 0.1	8.8 ± 0.2	6	9.2 ± 0.2	16.2 * ± 0.9	22	9.2 ± 0.1	15.8 ± 0.3	31	7.8 ± 0.1	14.8 ± 0.3	38	19.5 ± 0.2	28.8 * ± 0.2	16	7.8 ± 0.05	11.7 ± 0.08

A—in relation to the number of aspirated follicles (%); B—in relation to the number of recovered oocytes (%). *—statistically significant differences (*p* < 0.05).

**Table 2:** Effectiveness of the laparoscopic ovum pick-up (L-OPU) method among goats and ewes expressed per donor.

			Variant I (Goat)	Variant II (Goat)	Variant III (Goat)	Variant I to III in Total(Goat)	Variant IV (Goat)	Sheep
Number of ovarian follicles		Mean ± SD	9.8 * ± 4.2	6.6 ± 3.5	5.7 ± 5.4	6.5 ± 5.1	8.1 ± 4.1	4.7 ± 2.4
		Min	5	4	0	0	2	1
		Max	20	11	25	25	21	11
Number of aspirated follicles		Mean ± SD	9.6 * ± 4.3	6.5 * ± 3.4	5.5 ± 5.4	6.3 ± 5.1	7.8 ± 4.3	4.6 ± 2.3
		Min	4	1	0	0	2	0
		Max	20	9	25	25	21	11
Total number of recovered oocytes (%)		Mean ± SD	3.4 ± 1.2	3.7 ± 2.4	3.2 ± 3.4	3.3 ± 3.0	5.3 * ± 3.6	3.0 ± 1.8
		Min	2	0	0	0	2	0
		Max	5	9	18	18	17	9
Not qualified for IVM (Class IV)		Mean ± SD	0.2 ± 0.41	0.2 ± 0.44	0.2 ± 0.5	0.2 ± 0.5	0.2 ± 0.4	0.4 ± 0.6
		Min	0	0	0	0		0
		Max	1	1	2	2		2
Qualified for IVM Total (1 + 2 + 3)		Mean ± SD	3.2 ± 1.2	3.5 ± 2.4	3.0 ± 3.3	3.1 ± 2.9	4.5 * ± 1.7	2.62 ± 1.9
		Min	2	0	0	0	2	0
		Max	5	9	17	17	6	9
Morphology oocytes qualified for IVM	Class I (1)	Mean ± SD	1.2 ± 1.3	1.1 ± 2.2	1.3 ± 2.0	1.2 ± 1.9	2.3 * ± 1.2	1.1 ± 1.3
		Min	0	0	0	0	0	0
		Max	3	7	7	7	4	4
	Class II (2)	Mean ± SD	1.7 ± 1.9	1.8 ± 1.6	1.2 ± 2.3	1.4 ± 2.1	0.6 ± 1.1	1.29 ± 1.7
		Min	0	0	0	0	0	0
		Max	5	5	13	13	3	7
	Class III (3)	Mean ± SD	0.3 ± 0.5	0.6 ± 0.8	0.5 ± 0.9	0.5 ± 0.8	1.52 * ± 1.14	0.36 ± 0.9
		Min	0	0	0	0	0	0
		Max	1	2	2	2	3	4

SD—standard deviation; Min—minimum; Max—maximum; *—statistically significant differences (*p* < 0.05).

**Table 3:** Comparison of effectiveness of oocyte recovery using the ovum pick-up method in goats.

Method	Method Effectiveness (%)	Oocytes Qualified for In Vitro Maturation *	Author/Date
L-OPU **	71.8		Graff 1998 [15]
TUGA ***	61		
L-OPU	87.3		Baldassarre 2002 [38]
	82.1		
	73		
	83.7		
L-OPU	85.7	92.5	Baldassarre 2003 [1]
L-OPU	69.3	93.6	Cogniė 2003 [16]
	56.9	28.5	
L-OPU	56.25	46.4	Koeman 2003 [31]
	88.8	50.7	
L-OPU	87.6	72	Baldassarre 2007 [39]
L-OPU	81.2		Cox 2007 [17]
L-OPU	74.3	84	Souza 2013 [40]
L-OPU	47.4–86.5		Cordeiro 2014 [27]
L-OPU	91.2	85.1	Nor 2014 [41]
Post slaughter	73.0	57.2	Islam 2007 [42]

* in relation to the number of recovered oocytes (%), ** L-OPU—laparoscopic ovum pick-up, *** TUGA—transvaginal ultrasound-guided aspiration.

**Table 4:** Comparison of effectiveness of oocyte recovery using the ovum pick-up method in ewes.

Method	Method Effectiveness (%)	Oocytes Qualified for in Vitro Maturation *	Author/Date
L-OPU	79.5	60	Baldassarre 1996 [19]
	77.3	57	
L-OPU	67.9	83	Kühholzer 1997 [26]
L-OPU	55.5	65–70	Stangl 1999 [14]
L-OPU	56.2	85.7	Morton 2005 [43]
	38.8	81.8	
L-OPU	28.3–69.5		Rodríguez 2006 [20]
L-OPU	85.6		Cox 2007 [17]
L-OPU	51.7		Teixeira 2011[28]
L-OPU **	63.8	86.1	Wieczorek 2018 [13]

* in relation to the number of recovered oocytes (%), ** L-OPU—laparoscopic ovum pick-up.
